# Physician and the Machine: An Analysis of the Ethical Educational Use of Artificial Intelligence by Medical Students

**DOI:** 10.7759/cureus.117761

**Published:** 2026-10-11

**Authors:** Victor Hugo Policena de Jesus, Guilherme Cruvinel Jordão da Silveira, Pedro Enzo Camargo Luz, Luiza Letti Ferronatto, Thaís Bezerra Giovanini Fuscaldi, Enzo Villela de Faria, Kaio Albano Lopes, Igor Gifoni Aragão, Luisa Athayde Colnago, Alexandre Sampaio Rodrigues Pereira

**Affiliations:** 1 Medicine, Centro Universitário de Brasília, Brasília, BRA

**Keywords:** artificial intelligence, ethics, large language model, llms, medical education, medical students, medical training

## Abstract

Background

Artificial intelligence (AI) has been increasingly integrated into medical education, particularly through tools such as ChatGPT (OpenAI, California, US), Gemini (Google DeepMind, California, US), DeepSeek (Hangzhou DeepSeek Artificial Intelligence Basic Technology Research Co., Ltd., Hangzhou, China), and Microsoft Copilot (Microsoft Corp., Redmond, WA, US). Although these resources present the potential to expand access to information and assist in academic development, their use also raises ethical concerns related to the reliability of information, technological dependence, plagiarism, superficial learning, and potential impairment of clinical reasoning. This study aimed to investigate the perception of medical students regarding the use of AI in their academic training, emphasizing perceived ethical limits and practices considered ideal for its utilization.

Methods

This was a quantitative, descriptive, and cross-sectional study conducted with medical students using a structured questionnaire administered via a digital form.

Results

The sample consisted of 120 participants, of whom 99.2% (n=119) reported having previously used AI tools for academic purposes. It was observed that 73.3% (n=88) of the students use AI recurrently, primarily for summarizing content, researching medical information, simulating clinical cases, resolving questions, drafting academic texts, and planning studies. Most participants acknowledged the positive contribution of AI to learning, with 60.0% (n=72) stating that these tools contribute significantly and 36.7% (n=44) stating they contribute to a limited extent. However, students also identified relevant risks, especially the reduction of their own clinical reasoning, technological dependence, lack of information reliability, superficial learning, and academic plagiarism. Furthermore, 83.3% (n=100) stated that they did not receive adequate institutional guidance on the use of these tools, and 67.5% (n=81) demonstrated an interest in receiving further training on the ethical and pedagogical use of AI. No participant (0.0%, n=0) advocated for the prohibition of AI in academic contexts, while 67.5% (n=81) considered that its use should be regulated with ethical and pedagogical criteria.

Conclusions

AI already significantly integrates the academic routine of medical students and is perceived as a useful resource for learning, but it is still used in a scenario of insufficient institutional guidance. The findings reinforce the need for curricular guidelines, faculty training, and specific ethical education to promote the critical, responsible, and pedagogically appropriate use of AI in medical training.

## Introduction

Artificial intelligence (AI) has promoted a profound transformation in various sectors of society, and medical education is no exception. The use of AI-based systems, such as natural language models, machine learning algorithms, intelligent tutors, and clinical simulations, has reshaped how content is taught, assimilated, and applied in practice. In medical schools, this technology has consolidated itself as a promising pedagogical resource with the potential to personalize teaching, optimize academic performance, expand access to information, and facilitate the development of essential clinical and cognitive skills for future physicians [1].

Among the numerous advantages of using AI in medical education, its capacity to provide adaptive learning stands out, allowing the automatic adjustment of content according to the student's knowledge level and learning pace. Intelligent tutoring systems, for example, have been successfully used to teach complex topics, such as electrocardiogram interpretation, demonstrating positive results regarding usability, intention to use, and student engagement [2]. These systems enable students to practice repeatedly, receive real-time feedback, and build knowledge through gamified challenges and contextualized clinical simulations, resources proven to be effective for content retention and the stimulation of active learning [3].

Furthermore, natural language processing tools, such as ChatGPT (OpenAI, California, US), have been widely used by students to draft texts, organize studies, simulate clinical cases, and seek explanations for medical content. When properly guided, their use can develop skills such as clinical reasoning and problem-solving, bringing the student closer to a learning model centered on student protagonism. However, indiscriminate or uncritical use can lead to superficial learning, technological dependence, and failures in the scientific validation of the consulted information [4,5].

The growing presence of AI in medical training raises relevant ethical questions that are not yet sufficiently addressed in the curricula of Brazilian medical schools. Topics such as algorithmic bias, system reliability, the ethical use of sensitive data, patient autonomy, and responsibility for AI-assisted decisions must be systematically included in teaching programs. The concept of "AI literacy" has been widely advocated as a central axis to face this challenge, representing an approach that combines technical mastery of digital tools with a critical understanding of their ethical, social, and clinical impacts [6,7]. Integrating technical competence with critical reflection is essential, yet the Brazilian scenario remains incipient in this aspect, with a lack of practical experiences, pedagogical policies, and empirical studies to understand how medical students utilize and reflect on these tools in their academic daily lives.

The Brazilian context imposes specific challenges to the integration of AI in medical education. The majority of medical courses in Brazil do not systematically include the use of AI as an educational tool, nor do they offer formal spaces to discuss its ethical and social implications. The ethical use of AI requires not only awareness of its benefits and risks but also preparation to handle issues such as algorithmic bias, responsibility for automated decisions, the safe use of sensitive data, and impacts on the doctor-patient relationship [8]. In Brazil, this discussion gained normative backing only recently; during most of the period when this project was designed and data collected, the prevailing regulations did not mention AI [9]. In 2025, the National Education Council approved a new version of the National Curricular Guidelines for Undergraduate Medical Courses, explicitly incorporating emerging technologies - including AI - into national medical training [10]. Nevertheless, the lack of national data on how medical students perceive, use, and understand AI in their training exacerbates the gap between technological innovations and classroom reality [11,12].

Therefore, this descriptive cross-sectional study aims to investigate the perception of medical students regarding the use of AI in their training, gathering quantitative data on practices, perceptions, and ethical dilemmas to subsidize concrete actions for curricular improvement, pedagogical guidance, and institutional development.

## Materials and methods

Study design

This was a descriptive cross-sectional study, developed within an undergraduate research framework, aimed at investigating the perception of medical students regarding the use of AI in their academic training, with an emphasis on perceived ethical limits and practices considered ideal for the use of these tools in the context of medical school. The choice of a quantitative design is justified by the intention to collect, organize, and analyze objective data on the frequency of use, purposes, perceived benefits, ethical risks, and the evaluation of institutional guidance related to the use of AI. The descriptive nature is due to the purpose of characterizing the investigated phenomenon based on the students' own perceptions, without direct intervention by the researchers on the studied reality.

Study participants

The target population comprised students regularly enrolled in the medical course at Centro Universitário de Brasília (CEUB), Brazil. Medical students from different semesters who voluntarily agreed to participate in the study by accepting the informed consent form and who fully completed the questionnaire were included in the research. Participants who did not complete all stages of the form or who chose to interrupt their participation during the process were excluded, as foreseen in the initial project. The final sample consisted of 120 medical students whose responses were considered valid for analysis. All participants accepted the informed consent form and fully completed the questionnaire.

Data collection instruments

Data collection was carried out using a structured questionnaire (Appendix), developed specifically for this study, and administered in digital format via the Google Forms (Google LLC, California, US) platform. The instrument consisted of closed-ended, multiple-choice, and multiple-selection questions, organized into thematic blocks. The first block included sociodemographic and academic variables, such as age, gender, course semester, the existence of a scholarship or student financing, and familiarity with digital technologies. The second block addressed the practical use of AI in medical training, including the frequency of use, tools used, academic purposes, and perceived contribution to learning. The third block investigated the students' ethical perceptions, considering perceived risks, ethical training received, institutional guidance, impact on intellectual autonomy, and opinions on the ideal use of AI in the academic context. The question regarding perceived risks was formulated with the instruction to select up to three alternatives; however, due to limitations of the platform used, this cap was not automatically restricted during data collection. This structure aligns with the methodology planned in the initial project, which established three main axes: sociodemographic data, frequency and form of AI use, and perception of ethical limits and ideal use of these technologies in medical education.

Data collection procedures

Data collection was carried out using a structured questionnaire (Appendix), developed specifically for this study and validated through face validity by the research team. It was administered in digital format via the Google Forms platform. The instrument consisted of closed-ended, multiple-choice, and multiple-selection questions, organized into thematic blocks. The variable regarding familiarity with digital technologies was assessed based on the students' self-reported perception using a five-point Likert-type scale. Only after accepting the consent form could participants proceed to the questionnaire. Data collection was performed remotely through an electronic form, which enabled a wider reach among students and facilitated subsequent data organization.

Data analysis procedures

The obtained data were organized in an electronic spreadsheet and analyzed using simple descriptive statistics with the calculation of absolute and relative frequencies. Microsoft Excel (Version 16.0; Microsoft Corporation, Redmond, WA, US) was used for data tabulation and analysis. Categorical variables were presented in absolute numbers and percentages, allowing for the identification of the participants' profile, the frequency of AI use, the main academic purposes, perceived benefits, ethical risks identified, and the students' opinions regarding the need for regulation and institutional training. Multiple-selection questions were analyzed considering the number of selections per alternative and their proportion relative to the total number of participants. Due to the lack of restriction on the number of alternatives in the question about perceived risks, these data were treated as multiple-selection without a limit, so the percentages express the proportion of participants who recognize each risk, rather than a hierarchy of priority among them.

Ethical aspects

The study observed the ethical principles applicable to research involving human beings. Participation was voluntary, upon acceptance of the informed consent form, with a guarantee of anonymity, information confidentiality, and freedom to withdraw at any stage of the research. The study complied with ethical standards established by the Brazilian National Health Council (Resolution No. 466/2012) [9] and the General Data Protection Law, and it was approved by the institutional Research Ethics Committee of Centro Universitário de Brasília (CEUB) (CAAE: 91905225.3.0000.0023; Approval Protocol No. 7.911.071). No physical risks were anticipated for the participants. Possible moral risks, related to the exposure of information or discomfort when answering questions about the use of AI, were minimized through anonymity, data confidentiality, and the presentation of results only in grouped form. The expected benefits involved students' reflection on the ethical use of AI and the production of evidence to improve medical training in the face of current educational and technological challenges.

## Results

Sociodemographic and academic profile

The final sample consisted of 120 medical students. No incomplete responses were identified, allowing the full inclusion of the questionnaires in the analysis. There was a predominance of students aged 20 to 24 years, accounting for 65.0% (n=78) of the sample, and female subjects, who represented 62.5% (n=75). Most respondents were between their ninth and 12th semesters, corresponding to 53.3% (n=64) of the sample. Regarding student financing, 91.7% (n=110) declared not having a scholarship or financing. In terms of familiarity with digital technologies, 48.3% (n=58) reported high familiarity, and 15.8% (n=19) reported very high familiarity. The complete sociodemographic and academic profile of the participants is detailed in Table 1.

**Table 1:** Sociodemographic and academic profile of the study participants (n=120)

Variable	Category	Frequency (n)	Percentage (%)
Age (years)	20 to 24	78	65.0
	Others	42	35.0
Gender	Female	75	62.5
	Male	45	37.5
Course semester	9th to 12th	64	53.3
	Others	56	46.7
Student financing	No	110	91.7
	Yes	10	8.3
Digital familiarity	Moderate	39	32.5
	High	58	48.3
	Very high	19	15.8

Regarding the academic use of AI, 99.2% (n=119) of the participants reported having used some AI tool for academic purposes. Regarding the frequency of use, 37.5% (n=45) reported using AI tools "frequently," 20.8% (n=25) "almost always," and 15.0% (n=18) "always," totaling 73.3% (n=88) of students with recurrent AI use. The purposes of use were diverse, with summarizing content (77.5%, n=93), researching medical information (57.5%, n=69), and simulating clinical cases (56.7%, n=68) being the most cited (Figure 1).

**Figure 1:**
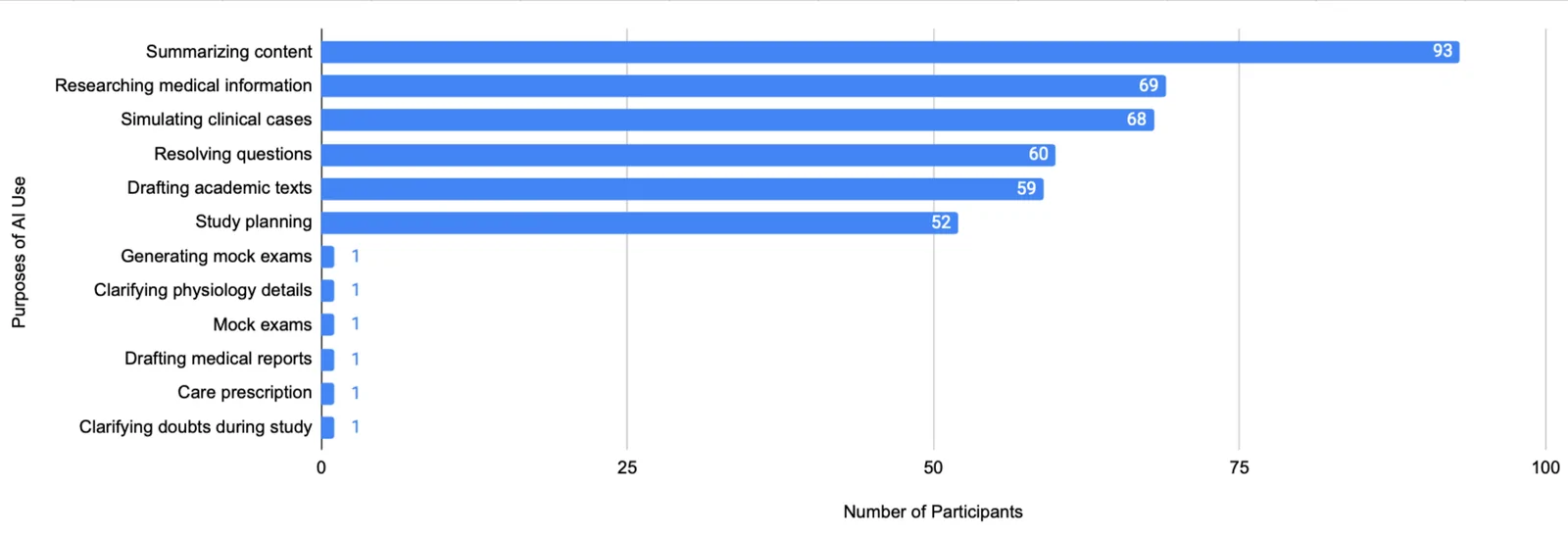
Purposes of artificial intelligence use among medical students (n=120)

The perception of AI's positive contribution to learning was high, with 60.0% (n=72) stating that these tools contribute "to a large extent" and 36.7% (n=44) recognizing a positive but limited contribution. When questioned about the use of AI in assessments or exams, 67.5% (n=81) declared never having used it, while 30.0% (n=36) affirmed having used it, and 2.5% (n=3) preferred not to answer.

Ethical perception regarding the use of AI

Only 17.5% (n=21) of the participants considered their training on AI ethics "very adequate," while 45.0% (n=54) rated it as "partially adequate" and 28.3% (n=34) as "nonexistent." The perception of institutional insufficiency was evident when asked about the guidance received to use these tools: 83.3% (n=100) stated they did not receive adequate guidance.

Regarding perceived risks, the reduction of their own clinical reasoning was the most cited (78.3%, n=94), followed by technological dependence (65.8%, n=79) and lack of information reliability (63.3%, n=76) (Figure 2).

**Figure 2:**
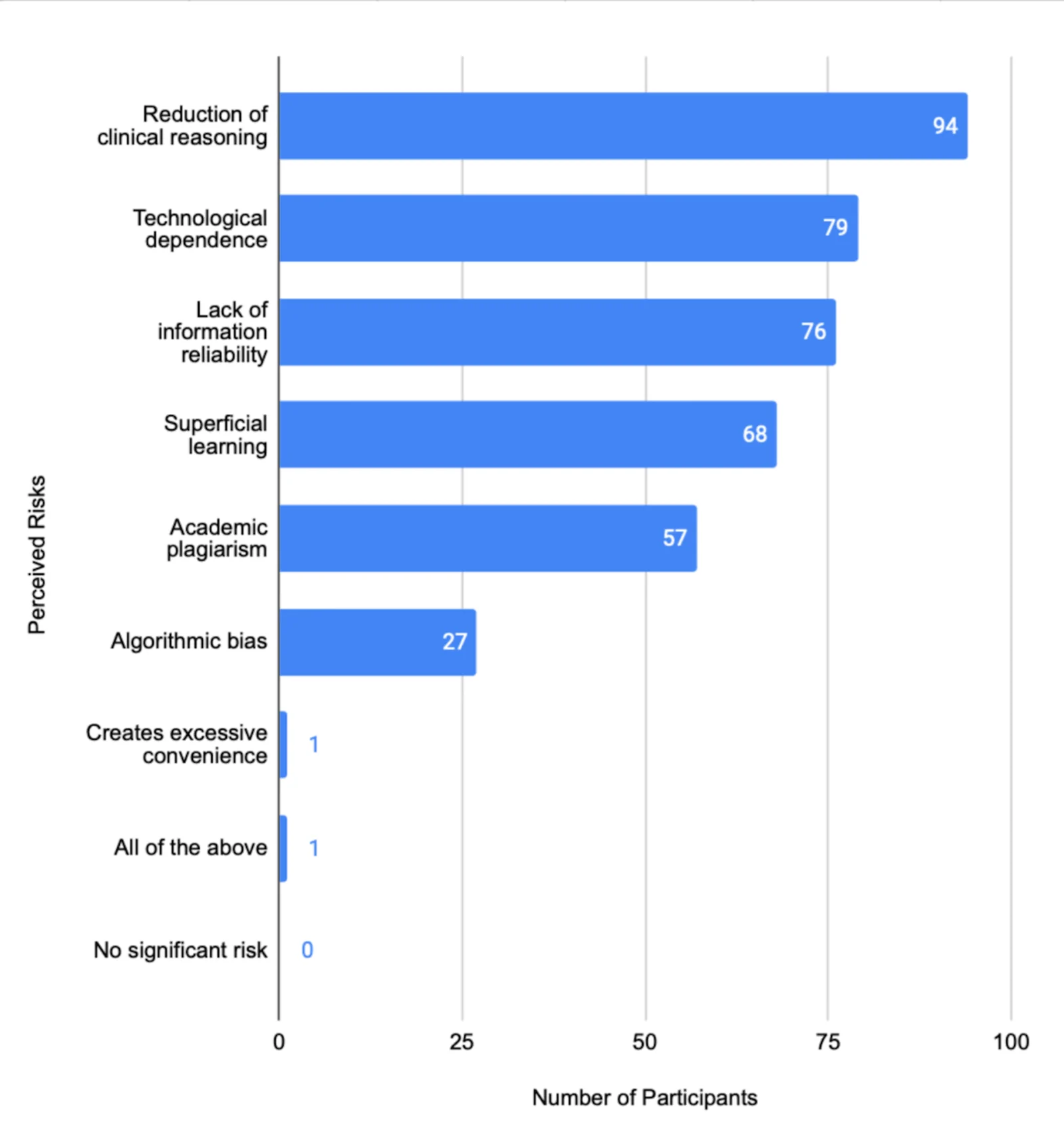
Perceived risks associated with the use of artificial intelligence in medical education (n=120)

Regarding intellectual autonomy, 64.2% (n=77) believed AI could negatively affect it depending on the use. Finally, 67.5% (n=81) indicated an interest in receiving more training on the ethical and pedagogical use of AI, and an identical percentage (67.5%, n=81) argued that its use in the academic context should be regulated with ethical and pedagogical criteria. No participant (0.0%, n=0) advocated for the prohibition of AI. The data on ethical perception are summarized in Table 2.

**Table 2:** Ethical perception, institutional guidance, and ideal academic use of artificial intelligence among medical students (n=120)

Variable	Category	Frequency (n)	Percentage (%)
Ethics training adequacy	Nonexistent	34	28.3
	Partially adequate	54	45.0
	Very adequate	21	17.5
	Do not know	11	9.2
Institutional guidance	Inadequate	100	83.3
	Partial	16	13.3
	Adequate	2	1.7
Ideal academic use	Regulated	81	67.5
	Free for students	35	29.2
	Prohibited	0	0.0

## Discussion

The predominance of students in the course's advanced periods is a relevant factor in interpreting the findings. These students face more complex academic and clinical demands, including case discussions, decision-making, and intensive content review. This context likely influences their recurrent use of AI for clinical reasoning and rapid information retrieval [11]. However, the almost universal use of AI among the participants contrasts sharply with the lack of formal institutional guidance, as the vast majority (83.3%, n=100) reported inadequate orientation. This discrepancy highlights a critical gap between student practice and institutional pedagogical response [12].

Students are utilizing AI not only for operational tasks, such as summarizing content, but also for activities directly linked to medical practice, including clinical case simulations and medical information research. While AI offers significant benefits in adaptive learning and personalized education [13,14], its unguided use poses risks to the development of clinical reasoning and intellectual autonomy. When AI is used to provide ready-made answers without critical mediation, it can compromise the construction of essential clinical competencies [4,5].

Interestingly, the students themselves demonstrated a high level of critical awareness. The most cited risk was the reduction of their own clinical reasoning (78.3%, n=94), followed by technological dependence (65.8%, n=79) and lack of information reliability (63.3%, n=76). They recognize the educational value of AI but demand clear criteria and regulations for its use, rather than a complete prohibition [15]. This aligns with international recommendations that emphasize the urgent need to integrate AI ethics into medical curricula, fostering a culture of responsible and transparent use rather than denial [7,8].

A major strength of this study is its novelty, as it provides pioneering empirical data on the ethical perceptions and practical use of AI by medical students in the Brazilian context, a topic with scarce national literature. By using a structured approach, it offers valuable insights that can directly inform the creation of new curricular guidelines.

This study has some limitations that should be acknowledged. The sample was predominantly composed of students from the ninth to 12th semesters. This predominance reduces the proportional representation of early-stage students and may influence the results, as advanced students have different clinical demands. Furthermore, because this is a study based on self-reported data collected through a digital questionnaire, the responses may be subject to perception, memory, or social desirability biases, especially regarding sensitive topics such as the use of AI in assessments. Additionally, students can use AI in creative ways, such as to remember acronyms in challenging lists. Mock exams can also be used by tutors; however, AI-generated exams could be less pedagogical and too perfect for proper assessment and inspiration [16]. Finally, AI hallucinations may interfere with proper AI-generated educational content, which means students should always critically assess the material.

The central debate should therefore shift from permitting or prohibiting AI to how to integrate these technologies ethically and transparently. Medical schools must develop institutional strategies that include clear guidelines on the use of AI in academic activities, faculty training, and the formal insertion of AI ethics into the curriculum. These tools should be utilized to support clinical reasoning rather than replace the student's intellectual autonomy [6,8].

## Conclusions

AI is already a concrete reality in medical education, widely utilized by students for various academic purposes. While students recognize the benefits of these tools for learning, they also demonstrate a critical awareness of significant risks, particularly the potential reduction of their own clinical reasoning, technological dependence, and academic plagiarism. However, a critical gap exists between the frequent use of these technologies and the lack of formal institutional guidance, leaving students without clear ethical and pedagogical parameters.

Rather than prohibiting AI, medical schools must actively regulate its use through clear institutional policies and the inclusion of digital literacy and ethics in the medical curriculum. Faculty training is essential to guide students in utilizing these tools as complementary resources that support, rather than replace, intellectual autonomy and clinical judgment. Ultimately, AI should serve as an auxiliary instrument integrated into a reflective educational environment, ensuring it enhances learning while preserving the fundamental human elements of medical training.

## Appendices

**Table 3:** Structure of the questionnaire applied in the study

Section	Question / Item	Response Options
Section 1: Sociodemographic Profile	What is your age?	Less than 20 years old; 20 to 24 years old; 25 to 29 years old; 30 years old or older
	Gender	Female; Male; Other; Prefer not to answer
	What semester of medical school are you currently in?	1st; 2nd; 3rd; 4th; 5th; 6th; 7th to 8th; 9th to 12th
	Do you have a scholarship or student financing?	Yes; No
	How do you evaluate your overall familiarity with digital technology?	Very low; Low; Moderate; High; Very high
Section 2: Use of Artificial Intelligence in Medical Training	Have you ever used any artificial intelligence tool for academic purposes?	Yes; No
	If yes, how often do you usually use AI tools to study?	Rarely; Occasionally; Frequently; Almost always; Always
	For what purposes do you use AI tools? (Check all that apply)	Summarizing content; Drafting academic works or texts; Resolving questions; Simulating clinical cases; Researching medical information; Study planning; Other
	Do you feel that the use of these tools contributes positively to your learning?	Yes, to a large extent; Yes, but to a limited extent; I am not sure; It does not contribute; It impairs my learning
	Have you ever used AI tools in assessments or exams (with or without authorization)?	Yes; No; Prefer not to answer
Section 3: Ethical Perception Regarding the Use of AI	How do you evaluate your training on artificial intelligence ethics in medical school?	Very adequate; Partially adequate; Nonexistent; I cannot evaluate
	In your opinion, what are the main risks of using AI in medical training? (Check up to 3)	Academic plagiarism; Technological dependence; Lack of information reliability; Superficial learning; Algorithmic bias; Reduction of own clinical reasoning; No significant risk; Other
	Do you consider that students receive adequate guidance from the institution on how to use these tools?	Yes; Partially; No; I cannot say
	Do you believe that the use of AI can negatively affect the student's intellectual autonomy?	Yes; No; Depends on the use; Never thought about it
Section 4: Ideal Training and Recommendations	Would you like to receive more training on the ethical and pedagogical use of AI in medical school?	Yes; No; Maybe
	In your opinion, AI should be:	Prohibited in academic contexts; Regulated with ethical and pedagogical criteria; Free for use according to the student's discretion; No opinion
